# *CYP2B6* c.983T>C polymorphism is associated with nevirapine hypersensitivity in Malawian and Ugandan HIV populations

**DOI:** 10.1093/jac/dku315

**Published:** 2014-08-20

**Authors:** Daniel F. Carr, Mas Chaponda, Elena M. Cornejo Castro, Andrea L. Jorgensen, Saye Khoo, Joep J. Van Oosterhout, Collet Dandara, Elizabeth Kampira, Francis Ssali, Paula Munderi, David G. Lalloo, Robert S. Heyderman, Munir Pirmohamed

**Affiliations:** 1 Department of Molecular and Clinical Pharmacology,University of Liverpool, Liverpool, UK; 2 Malawi–Liverpool–Wellcome Trust Clinical Research Programme, College of Medicine, University of Malawi, Malawi; 3 Department of Biostatistics, University of Liverpool, Liverpool, UK; 4 Dignitas International, Zomba, Malawi; 5 Division of Human Genetics, University of Cape Town, Cape Town, South Africa; 6 Department of Pathology, College of Medicine, Blantyre, Malawi; 7 Joint Clinical Research Centre, Kampala, Uganda; 8 UVRI/MRC Uganda Research Unit on AIDS, Entebbe, Uganda; 9 Liverpool School of Tropical Medicine, Liverpool, UK

**Keywords:** pharmacogenetics, adverse drug reactions, Stevens–Johnson syndrome, antiretroviral

## Abstract

**Background:**

Nevirapine, an NNRTI used in HIV treatment, can cause hypersensitivity reactions in 6%–10% of patients. In the most serious cases (1.3%) this can manifest as Stevens–Johnson syndrome (SJS) or toxic epidermal necrolysis (TEN).

**Methods:**

DNA samples were obtained and analysed from a total of 209 adult patients with nevirapine hypersensitivity (57 from a prospective cohort and 152 routine clinic patients) and compared with 463 control patients on nevirapine without any hypersensitivity. The case group included 70 patients with SJS/TEN. All individuals were genotyped for two SNPs in the *CYP2B6* gene [c.516G>T (CYP2B6*9) and c.983T>C (CYP2B6*18)] using the TaqMan real-time genotyping platform. The replication cohort comprised 29 controls and 55 nevirapine hypersensitive patients, including 8 SJS/TEN cases.

**Results:**

An association between the *CYP2B6* c.983T>C polymorphism and nevirapine-induced SJS/TEN was observed. In the SJS/TEN group, 30% of individuals possessed at least one c.983T>C versus 16% in the tolerant group [*P* = 0.006; OR (95% CI) 2.24 (1.27–3.94)]. This association was not significant in the replication cohort [*P* = 0.075; OR (95% CI) 4.33 (0.80–23.57)]. Combined analysis resulted in an OR of 2.52 (95% CI 1.48–4.20; *P* = 0.0005) for the association of c.983T>C with SJS/TEN. No association was observed for *c.983T>C* with other hypersensitivity phenotypes and for *CYP2B6* c.516G>T with any hypersensitivity phenotypes.

**Conclusions:**

Our data show an association between the c.983T>C polymorphism and nevirapine-induced SJS/TEN. *CYP2B6* c.983T>C has a frequency of 5%–10% in a variety of African populations, but is not observed in Caucasians, thus representing an ethnic-specific predisposing factor.

## Introduction

Nevirapine is a potent NNRTI used in the treatment of HIV, now largely used in developing countries. Individuals taking nevirapine have a 6%–10% risk of developing a hypersensitivity reaction (HSR).^1,2^ This can manifest clinically as hypersensitivity syndrome (HSS) (fever, skin rash), severe blistering skin rashes, such as Stevens–Johnson syndrome (SJS) and toxic epidermal necrolysis (TEN),^3^ and hepatotoxicity.^4^ The risk of nevirapine HSR is significantly higher in individuals who start nevirapine at higher CD4+ cell counts (>400 cells/mm^3^ and >250 cells/mm^3^ for men and women, respectively).^5^

Other drugs metabolized by P450 enzymes associated with HSRs, including carbamazepine^6^ and abacavir,^7^ have shown strong associations with human leucocyte antigen (HLA) alleles in the major histocompatibility complex region of chromosome 6. Nevirapine HSRs have also been linked with HLA alleles including HLA-DRB1*0101,^8,9^ *HLA-Cw8*,^10^ HLA-B*35: 05^11^ and, most recently, HLA-C*04: 01,^12^ with the HLA predisposition for liver injury different from that of skin injury.^13^ However, these associations are not as strong as those observed with other drugs, such as abacavir, and vary with both ethnicity and phenotype.

Nevirapine is mainly metabolized to 8-hydroxynevirapine by the hepatic cytochrome P450 enzymes CYP2B6 and, to a lesser extent, CYP3A4.^14^ Despite its extensive use in WHO-recommended regimens in Africa, the effect of polymorphisms in genes encoding nevirapine-metabolizing enzymes has not been extensively studied. CYP2B6 is characterized by wide inter-individual variability in expression and activity in human livers.^15,16^ This variability is due, in part, to the effects of genetic polymorphisms. One specific variant in exon 4 (c.516G>T), encoding a non-synonymous amino acid substitution (p.G172H) (rs3745274) (CYP2B6*6/9), is associated with a significant loss of function.^15–18^ Studies have shown that the variant T allele is associated with higher plasma concentrations in adult populations of both Western European^19^ and sub-Saharan African descent.^20^ Recently, c.516G>T was reported to be associated with nevirapine-induced cutaneous adverse events in black and white populations.^13^ This association was stronger when the c.516G>T genotype was combined with carriage of the HLA-Cw*04 allele.^13^ The *c.516G>T* polymorphism has also been linked with nevirapine-induced neuropsychological toxicity,^19^ but not with nevirapine-induced hepatotoxicity.^13,21^

Recent *in vitro* studies functionally characterizing *CYP2B6* alleles showed that the CYP2B6*18 polymorphism defined by c.983T>C (rs rs28399499) was enzymatically inactive,^22^ while other polymorphisms (for example, *c.516G>T*) did demonstrate some, albeit reduced, enzymatic activity. A small pharmacokinetic/pharmacogenetic sub-study in our Malawian cohort showed that, whilst CYP2B6*18 had an effect on nevirapine plasma concentrations, there was no association between nevirapine plasma concentrations *per se* and any hypersensitivity phenotype.^23^ However, an association has been demonstrated between nevirapine-induced hepatotoxicity in a Mozambican cohort and the c.983T>C polymorphism.^24^ The frequencies of both the c.516G>T and c.983T>C polymorphisms can differ between different ethnicities. In particular, the c.983T>C is almost exclusively found in populations of African ancestry.^25^

The primary objective of this study was to determine, in a large patient cohort, whether c.516G>T and c.983T>C polymorphisms are predisposing factors for nevirapine hypersensitivity in a Malawian HIV-infected adult population. Secondly, we aimed to investigate whether carriage of HLA-C*04: 01 in combination with variants of *CYP2B6* increases the risk for nevirapine hypersensitivity.

## Patients and methods

### Patients

#### Discovery cohort

Nevirapine-exposed hypersensitive cases and controls were recruited from the outpatient clinic at the Queen Elizabeth Central Hospital between March 2007 and December 2008 as previously described.^12^ Briefly, 1117 patients within the prospective study cohort were enrolled at the start of the standard first-line ART regimen at the time (stavudine/lamivudine/nevirapine) and followed for 6 months. Of these, 57 patients developed an HSR as defined previously.^12^ Additionally a further 149 nevirapine hypersensitive patients were recruited at the time of reaction from the same centre separately to the prospective study and 28 were identified retrospectively from patient records. Thus a total of 234 nevirapine hypersensitive patients were identified. The hypersensitivity phenotypes and total numbers are summarized as follows: (i) nevirapine-induced rash (NIR) (*n* = 83), including patients with widespread maculopapular rash without systemic manifestations and getting worse on treatment continuation; (ii) HSS (*n* = 39), including patients with widespread rash and systemic manifestations such as fever, cough or abnormal liver function tests; (iii) SJS (*n* = 75), including extensive rash with the involvement of at least two mucous membranes or blistering eruptions affecting <10% of the body surface area; (iv) TEN (*n* = 4), including blistering rash affecting more than 30% of the body surface area and mucous membrane involvement as per SJS^26^ (blistering of 10%–30% of the body surface area was termed overlap syndrome); and (v) drug-induced liver injury (DILI) (*n* = 36), manifesting with clinically evident jaundice and abnormal ALT (>40 IU/L).

Three patients presented with both SJS/TEN and DILI. Causality assessment was undertaken using the Naranjo causality assessment tool^27,28^ and reviewed independently by a dermatologist.

Controls were patients in the prospective cohort who had been on nevirapine for at least 6 months without developing any features of hypersensitivity.

#### Replication cohort

An additional 24 cases presenting with the hypersensitivity phenotype according to the original criteria were identified retrospectively from the Queen Elizabeth Central Hospital, Blantyre, Malawi, after the conclusion of the initial recruitment phase (December 2008).

Thirty-two nevirapine hypersensitivity cases and 31 tolerant controls were identified retrospectively in Uganda from the DART study cohort.^29^ Cases were defined according to available patient records and subsequently categorized into the sub-phenotypes previously described.

### Ethics

The study received full ethics approval from the Liverpool School of Tropical Medicine Research Ethics Committee (Liverpool, UK), the College of Medicine Research and Ethics Committee, University of Malawi (Blantyre, Malawi) and the Uganda National Council for Science and Technology. All patients gave their written informed consent. All patients who met the criteria for a case had nevirapine withdrawn in accordance with Malawian National Treatment Guidelines. Local ethics approval was obtained for the DART study as previously described^29^ with subsequent ethics approval for a pharmacogenetic sub-study also obtained.^30^

### DNA extraction and genotyping

DNA was extracted from whole blood using a salt precipitation protocol.^31^ Sufficient DNA was available from a total of 672/1294 (52%) individuals in the discovery cohort (463 controls and 209 hypersensitivity cases). For the replication cohort sufficient DNA was available from a total of 82/87 (94%) (29 controls and 53 cases). Genomic DNA was extracted from 5 mL of whole blood collected using the Oragene DNA Sampling kit (DNAGenotek, Ontario, Canada) with the Chemagic Magnetic Module (MSM) 1 system as per the manufacturer's protocol (Chemagen Biopolymer-Technologie AG, Baesweiler, Germany). All samples were genotyped for the c.516G>T and c.983T>C validated TaqMan Drug Metabolism Genotyping Assays (Applied Biosystems, Foster City, CA, USA).

PCRs (5 μL) consisted of 1× Taqman Universal PCR master mix (Applied Biosystems), 1× assay mix [unlabelled PCR primers and TaqMan minor groove binding probes (FAM™ and VIC^®^-labelled)] and 20 ng of DNA. PCR was performed using an Applied Biosystems 7900HT real-time PCR system (Applied Biosystems). The following cycling conditions were used: 95°C for 1 min followed by 40 cycles of 92°C for 15 s and 60°C for 1 min. Allelic discrimination analysis and genotype calls were made with the ABI 7900HT Sequence Detection System (Applied Biosystems). Ten percent of samples were duplicated in order to determine genotype concordance. Genotype call rates of >95% and a Hardy–Weinberg equilibrium *P* value >0.025 were also applied as cut-offs for both SNPs.

### Statistical analysis

Statistical analysis was undertaken using SPSS 16.0 software, apart from case–control analyses, which was carried out using Haploview 4.1 software (www.broad.mit.edu/mpg/haploview/). Association between each non-genetic variable in turn (gender, age, BMI, CD4+ cell count) and nevirapine-induced hypersensitivity risk was assessed using binary logistic regression models. To test for association between each nevirapine-induced hypersensitivity phenotype (all HSRs, NIR, HSS, SJS/TEN, DILI) and each SNP in turn, two binary regression models were fitted, The first (base model) included all non-genetic variables found statistically significant in the univariate analyses (*P* < 0.05). The second (SNP model) was the same as the base model but also included a covariate to represent the SNP of interest. An additive effect of the variant allele was assumed. Homozygote wild-type was coded ‘0’, heterozygote ‘1’ and homozygote variant allele ‘2’. For analysis of composite genotype (number of c.516T and c.983C alleles), an additive effect was assumed and the composite genotype coded according to the number of variant alleles carried.

The likelihood ratio test was used to compare the base model with the SNP model and thus determine whether association between the SNP and outcome was statistically significant. Case–control comparison of allele counts was also undertaken using a χ^2^ test. For all analyses, both a *P* value and the false discovery rate (FDR) were calculated.

Meta-analysis of our study data with previously published data was undertaken using StatsDirect v2.6.8 (StatsDirect Ltd, Altrincham, UK) using a random effects model.

## Results

A total of 672 patients where DNA was available were genotyped as the discovery cohort study. Demographic data for both nevirapine tolerant and intolerant patients are shown in Table 1. Females accounted for 62.7% of the adverse event group compared with 55.7% in the tolerant group, and no significant association was found between gender and nevirapine-induced hypersensitivity risk (*P* = 0.09). Further, mean age ± SD (37.4 ± 9.4 versus 36.2 ± 9.8 years) and mean BMI ± SD (21.0 ± 4.0 versus 20.6 ± 3.6 kg/m^2^) were not significantly different between the adverse event and tolerant groups (*P* = 0.15). Mean CD4+ cell count ± SD in the adverse event group (294 ± 208 cells/mm^3^) was significantly higher than in the tolerant group (174 ± 144 cells/mm^3^) (*P* = 2.8 × 10^−13^ in logistic regression analysis). Only CD4+ cell count was therefore carried over as a covariate into the logistic regression base model.

**Table 1.** Demographic and phenotypic data for the nevirapine discovery and replication cohorts

	Discovery cohort (*n* = 672)			Replication cohort (*n* = 82)		
	cases (*n* = 209)	controls (*n* = 463)	*P* value	cases (*n* = 53)	controls (*n* = 29)	*P* value
Gender	M: 78 (37%)	M: 205 (44%)		M: 22 (42%)	M: 8 (28%)	
	F: 131 (63%)	F: 258 (56%)	0.09	F: 31 (58%)	F: 21 (72%)	0.005
Age (years), mean ± SD	37.4 ± 9.4	36.2 ± 9.8	0.11	38.0 ± 8.98	37.9 ± 6.6	0.010
BMI (kg/m^2^), mean ± SD	21.0 ± 4.0	20.6 ± 3.6	0.15	21.3 ± 6.5	22.2 ± 7.3	0.025
CD4+ count (cells/mm^3^), mean ± SD	294 ± 208	174 ± 144	<0.0001	145 ± 125	79 ± 54	0.002

M, male; F, female.

*P* values derived from binary logistic regression analysis.

The phenotypes of the 209 nevirapine hypersensitive patients included in the discovery cohort genotype analysis were categorized as 76 NIR, 36 HSS, 67 SJS/TEN and 27 DILI with 3 of the patients recorded as having SJS/TEN and DILI. Genotyping of both polymorphisms (c.516G>T and c.983T>C) passed the pre-determined call rate (95.1% for c.516G>T and 99.4% for c.983T>C) and Hardy–Weinberg equilibrium *P* value (*P* = 0.059 for c.516G>T and 0.89 for c.983T>C) quality control thresholds. The *CYP2B6* genotype distributions and allele frequencies in the study cohort (*n* = 672) are summarized in Table 2.

**Table 2.** Association of CYP2B6 polymorphisms with nevirapine-induced HSR phenotypes in the discovery cohort

	*CYP2B6* c.516G>T							*CYP2B6* c.983T>C						
	*n*	MAF	*P* value^a^	genotype frequency			*P* value^b^	*n*	MAF	*P* value^a^	genotype frequency			*P* value^b^
				GG	GT	TT					TT	TC	CC	
Tolerant	428	0.46	—	0.25	0.57	0.18	—	460	0.08	—	0.84	0.15	0.01	—
All HSRs	209	0.41	0.106	0.36	0.45	0.19	**0**.**022**	208	0.12	0.076	0.77	0.13	0.00	**0**.**037**
NIR	75	0.41	0.275	0.33	0.51	0.16	0.337	76	0.09	0.765	0.83	0.19	0.00	0.412
HSS	36	0.34	0.615	0.47	0.36	0.17	**0**.**036**	36	0.12	0.245	0.78	0.19	0.03	0.386
SJS/TEN^c^	70	0.46	0.924	0.36	0.43	0.24	0.276	70	0.15	**0**.**014**	0.70	0.30	0.00	0.052
DILI^c^	30	0.35	0.094	0.43	0.43	0.13	0.301	29	0.07	0.673	0.86	0.14	0.00	**0**.**047**

MAF, minor allele frequency.

*P* values <0.05 are shown in bold.

^a^*P* value from χ^2^ test of association between allele count and phenotype.

^b^*P* value from likelihood ratio test comparing logistic regression models both with and without covariate representing SNP (adjusted for CD4+ cell count).

^c^Three patients presented with both SJS/TEN and DILI phenotypes.

When undertaking χ^2^ tests of association between allele counts for each SNP and each nevirapine hypersensitivity phenotype in turn, no statistically significant associations were found (FDR > 0.05 for all analyses) (Table 2). Furthermore, there were also no statistically significant associations in the logistic regression analyses (FDR > 0.05 for all analyses) (Table 2). Case–control analysis by logistic regression of a composite CYP2B6 genotype based on the total number of minor alleles carried (Table 3) also failed to show any significant associations with any of the hypersensitivity phenotypes (FDR > 0.05).

**Table 3.** Association of *CYP2B6* composite genotype (total number of c.516T and c.983C alleles) with nevirapine-induced HSR phenotypes in the discovery cohort

	*n*	Frequency of *CYP2B6* minor allele carriage (c.516G≥T and c.983T≥C)				*P* value^a^
		0 alleles	1 allele	2 alleles	3 alleles	
Tolerant	425	0.20	0.52	0.28	<0.01	—
All HSRs	207	0.27	0.42	0.31	0.01	0.066
NIR	75	0.29	0.40	0.31	0.00	0.091
HSS	36	0.33	0.39	0.28	0.00	0.220
SJS/TEN^b^	70	0.17	0.40	0.37	0.04	0.062
DILI^b^	29	0.38	0.41	0.21	0.00	0.244

^a^*P* value from likelihood ratio test comparing logistic regression models both with and without covariate representing allele count (adjusted for CD4+ cell count).

^b^Three patients presented with both SJS/TEN and DILI phenotypes.

Since the association between c.983G>T and SJS/TEN was the only significant observation where an increased frequency of the functional minor allele was observed, an analysis of carriage frequency incorporating a replication cohort was undertaken (Table 4). In the discovery cohort, c.983C carriage was significantly associated with SJS/TEN risk [*P* = 0.006, OR = 2.24 (95% CI 1.27–3.94)]. The association was further strengthened when combined with the replication cohort [*P* = 0.0005.OR = 2.52 (95% CI 1.48–4.20); FDR = 0.015].

**Table 4.** Association of CYP2B6*18 polymorphism carriage with nevirapine-induced SJS/TEN in the Malawian discovery cohort and the sub-Saharan African replication cohort

Cohort	Controls		SJS/TEN		*P* value	OR (95% CI)
	*n*	CYP2B6*18 carriage	*n*	CYP2B6*18 carriage		
Discovery	460	74 (0.16)	70	21 (0.30)	0.006	2.24 (1.27–3.94)
Replication	29	3 (0.10)	8	4 (0.50)	0.075	4.33 (0.80–23.57)
Combined	489	77 (0.16)	78	25 (0.32)	0.0005^a^	2.52 (1.48–4.20)

*P* values and OR (95% CI) determined from χ^2^ test.

^a^FDR < 0.05.

Meta-analysis of the c.983T>C polymorphism carriage and SJS/TEN of our data and the only previous report that investigated an association with nevirapine-induced SJS/TEN^32^ produced a combined OR of 2.61 (95% CI 1.61–4.24) (data not shown).

Data relating to HLA-C allelotypes from our previous analysis^12^ was available for 124 controls and 32 SJS/TEN patients from the discovery cohort. Carriage of both the HLA-C*04: 01 and c.983T>C allelic variants together was not significantly associated with SJS/TEN (*P* = 0.16) with a frequency of co-occurrence of 0.12 in controls and 0.21 in SJS-TEN.

## Discussion

In this large study in Malawian and Ugandan adults treated with nevirapine, we have been able to show an association between nevirapine-induced SJS/TEN and the c.983T>C polymorphism. The carriage frequency in nevirapine control individuals was 16% compared with 32% in those with nevirapine-induced SJS/TEN (*P* = 0.0005, FDR = 0.015) (Table 4). This association was not observed with any other nevirapine-induced hypersensitivity phenotype.

The c.983T>C polymorphism is non-synonymous, encoding an isoleucine to threonine amino change at codon 328 in exon 7. Previous studies have suggested that the rs2839949 C allele is present at a frequency of 4.4% in African–Americans and 6.6% in a Ghanaian cohort, but is absent in Caucasians.^33^ *In vitro* studies of the recombinant c.983C allele have demonstrated undetectable levels of expression and enzymatic activity,^33^ while *in vivo* studies have shown that it acts as a determinant of nevirapine plasma concentrations.^34,35^ It could be speculated that the associated risk of SJS/TEN with the C allele is a result of increased circulating parent compound or possibly biotransformation of nevirapine being pushed down an alternative pathway.

The c.516G>T polymorphism has previously been associated with increased plasma nevirapine concentration in a number of studies of patients of varied ethnicity^19,34,36,37^ as well as cutaneous adverse events in both white and black populations.^13^ Our data appear to contradict this; however, it should be noted that our data are derived mainly from a relatively homogeneous Malawian population whilst previous studies described findings in a self-reported black population where there was more ethnic heterogeneity. Further investigation evaluating whether there is any population stratification would be required to resolve this issue.

The CYP2B6*4 polymorphism (c.785A>G; rs2279343) that, in combination with the c.516 G>T (CYP2B6*9) SNP, defines the CYP2B6*6 allele was also analysed in our cohort (data not shown). The *CYP2B6* c.785A>G SNP was found to be in high linkage disequilibrium (*r*^2^ = 0.96) with the CYP2B6*9 defining c.516G>T SNP and so it was felt likely that CYP2B6*6 (c.516T and c.785G) would not be significantly associated with nevirapine hypersensitivity (as per our findings for *c.516G>T*).

One interesting observation of this study is the identification of three individuals (all SJS/TEN) who are homozygous for c.516T and heterozygous for c.983C and two individuals (both nevirapine tolerant) who are heterozygous for c.516G>T and homozygous for c.983C (shown as carriers of 3 alleles in Table 3). Thus, all five appear to carry a previously unreported haplotype whereby c.516T and c.983C are carried on the same chromatid. However, a lack of statistical power prevents any specific conclusions being drawn as to whether the haplotype may be a significant risk factor for SJS/TEN.

Within our patient group, CD4+ T cell count was a statistically significant risk factor for nevirapine hypersensitivity (Table 1) with the mean CD4+ T cell count being higher in patients experiencing adverse events (294 versus 174 cells/mm^3^). This observation is consistent with other studies that have described CD4+ cell count as a risk factor for nevirapine-induced hypersensitivity.^38^ The implication from this is that, in an HIV patient treated with nevirapine, a partially functioning immune system is needed to mount an immune response. We have previously shown that HLA-C*04: 01 acts as a risk factor for nevirapine-induced SJS/TEN consistent with the role of the immune response in the pathogenesis of nevirapine hypersensitivity. Here we found no significant association between co-carriage of HLA-C*04: 01 and the c.983T>C allele, and nevirapine-induced SJS/TEN, but this may have been because of lack of power.

Previous studies in black African populations have demonstrated that both the c.516G>T^23,34^ and c.983T>C polymorphisms affect plasma nevirapine concentrations. Both polymorphisms have been shown to be associated with hypersensitivity in a number of studies. Although some evidence does exist,^37–40^ there does not seem to be a compelling direct functional association between plasma concentrations and the risk of nevirapine hypersensitivity. It is possible that local concentrations in the skin may be more important. CYP2B6 is expressed in keratinocytes in particular within the epidermis.^41^ Thus, in individuals with decreased functional activity of CYP2B6, higher skin concentrations may be attained, which could result in dermal immune activation. The lack of association with HSS may be a reflection of the lack of power, while the lack of association with NIR may be due to the fact that causality is more difficult to assess with the milder skin reactions.

A limitation of our study is the size of the replication cohort, which was smaller than the discovery cohort and therefore lacked the statistical power to truly replicate the association observed between c.983T>C and nevirapine-induced SJS/TEN. However, nevirapine hypersensitivity is a rare phenotype and it was difficult to identify a larger number of patients. The replication cohort also consisted of both Malawian and Ugandan patients, which may introduce some population stratification. However, our data show that the frequency of c.983T>C polymorphism in the overall combined Malawian discovery and replication patients (0.18) was comparable to that observed in the Ugandan patients (0.15), and to that reported in a Mozambican population (0.14).^32^ Although genetic differences do exist between these African cohorts, it would appear from our study that *CYP2B6* c.983T>C is likely to be generalizable across other sub-Saharan-African populations.

In conclusion, our data suggest that the c.983T>C polymorphism, but not c.516G>T, is a risk factor for nevirapine-induced SJS/TEN in HIV-infected African adults from two different populations. Further studies are needed to determine the role of metabolic pathways in the predisposition to severe skin reactions, and how this leads to immune activation locally within the skin.

## Funding

This work was supported by a 3 year Wellcome trust training fellowship WT078857MA awarded to M. C. and administered through the University of Liverpool. The Malawi–Liverpool–Wellcome Trust Clinical Research Programme is funded through a Core Programme Grant award from the Wellcome Trust. M. P. is an NIHR Senior Investigator. Additionally the DART study was supported by the UK MRC (grant number G0600344), the UK Department for International Development and the Rockefeller Foundation. GlaxoSmithKline, Gilead Sciences, Boehringer-Ingelheim and AbbVie donated drugs for DART.

## Transparency declarations

The authors have no conflicts of interest to declare. Boehringer Ingelheim Pharmaceuticals, who donated nevirapine to the DART study, had no role in the design, analysis or interpretation of the results in this study, but were given the opportunity to review the manuscript for medical and scientific accuracy, as well as intellectual property considerations.
